# FPR2 participates in epithelial ovarian cancer (EOC) progression through RhoA-mediated M2 macrophage polarization

**DOI:** 10.1186/s13048-021-00932-8

**Published:** 2021-12-20

**Authors:** Xiaohui Xie, Juan He, Qiong Wang, Yaqiong Liu, Weiwei Chen, Kun Shi

**Affiliations:** Department of Gynaecology and Obstetrics, Guangzhou Women and Children’s Medical Center, Guangzhou Medical University, Guangzhou, China

**Keywords:** Epithelial ovarian cancer, FPR2, RhoA, Macrophage

## Abstract

**Background:**

In our previous study, we found that formyl peptide receptor 2 (FPR2) promoted the invasion and metastasis of epithelial ovarian cancer (EOC) and could be a prognostic marker for EOC. In this study, we aimed to study the possible mechanism of FPR2 in promoting EOC progression.

**Methods:**

EOC cell lines with ectopic FPR2 expression and knockdown as well as their control cell lines were established, and the expression change of RhoA in each cell line was evaluated by real time quantitative polymerase chain reaction (RT-qPCR) and Western blot. Wound healing and Transwell assays were performed to detect the migratory ability of EOCs affected by FPR2 and RhoA. The supernatant of each EOC cell line was used to coculture with macrophages, and then we tested M1 and M2 macrophage biomarkers in the supernatants by flow cytometry. The THP-1 cell line was also induced to differentiate into M1 and M2 macrophages, and FPR2 and RhoA expression in each macrophage cell line was detected by RT-qPCR and Western blot. A tumour xenograft model was established with SKOV3 and SKOV3^−shFPR2^ cell lines, and tumour volumes and weights were recorded.

**Results:**

RhoA expression was significantly increased in EOCs along with the overexpression of FPR2, which showed a positive correlation by Pearson correlation analysis. Ectopic FPR2 expression contributes to the migratory ability of EOCs, and a RhoA inhibitor (C3 transferase) impairs EOC migration. Furthermore, FPR2 stimulated the secretion of Th2 cytokines by EOCs, which induced macrophages to differentiate to the M2 phenotype, while a RhoA inhibitor stimulated the secretion of Th1 cytokines and induced macrophages to differentiate to the M1 phenotype. Moreover, compared with M1 macrophages and THP-1 cells, FPR2 and RhoA expression was significantly upregulated in M2 macrophages.

**Conclusion:**

FPR2 stimulated M2 macrophage polarization and promoted invasion and metastasis of ovarian cancer cells through RhoA.

## Introduction

Ovarian cancer is one of the most lethal gynaecological malignancies in the world. Seventy-five percent of patients were first diagnosed with ovarian cancer in the advanced stage because of a lack of clinical symptoms. Moreover, ovarian cancer is characterized by rapid progression, poor prognosis and high rates of recurrence and metastasis. At present, in addition to classical treatments, including operation and postoperative chemotherapy, neoadjuvant chemotherapy, immunotherapy and molecular targeted therapy are recommended. Nevertheless, the five-year survival rate for ovarian cancer has not improved in the past 20 years, fluctuating between 30 and 35% [1, 2].

Formyl peptide receptor 2 (FPR2) has been identified as a member of the G protein-coupled chemoattractant receptor (GPCR) family. It is a seven-transmembrane receptor with 351 amino acids, and its gene is located on human chromosome 19q13.3-q13.4 [3]. FPR2 is a multifunctional receptor that is associated with diverse pathophysiological processes, such as inflammation, cancer, amyloidosis, neurodegenerative diseases, wound healing, diabetes and AIDS [4]. In a previous study, FPR2 was shown to be overexpressed in ovarian cancer tissues and to be located on the cell membrane by IHC. Additionally, knocking down FPR2 inhibited the invasion and migration of ovarian cancer cells, potentially indicating that FPR2 plays a key role in cancer cell metastasis. Moreover, FPR2 is closely related to the clinical prognosis of ovarian cancer patients [5]. Small GTPases of the Rho family are involved in FPR signalling [6, 7]. The most noticeable member of the Rho family is RhoA, which acts as a “molecular switch” to activate downstream signalling pathways. Here, we investigated whether RhoA is involved in the role of FPR2 in ovarian cancer invasion and migration.

In recent years, the tumour microenvironment, which is composed of tumour cells, fibroblasts, immune cells, endothelial cells, extracellular matrix, cytokines, etc., has been considered vital in malignant development. Macrophages are major tumour-infiltrating immune cells that are associated with tumour progression and account for almost 50% of the total immune cells in the tumour microenvironment [8]. Blood-derived monocytes in tumour tissues differentiate into macrophages and then further develop into the M1 or M2 phenotype depending on different conditions. When stimulated by Th1 cytokines, such as IFN-γ, LPS, TNF-α, reactive oxygen species (ROS), IL-1, and IL-12, infiltrating macrophages differentiate into the M1 phenotype, which shows a cytotoxic effect, promotes immune activation and inhibits malignant progression. When stimulated by Th2 cytokines, including M-CSF, GM-CSF, IL-4, IL-10, and TGF-β, macrophages differentiate into the M2 phenotype, which is equipped with the functions of immune suppression, facilitating tumour progression, cell proliferation and angiogenesis [9]. M2 macrophages have been suggested to be able to play a role in ovarian cancer progression, and the larger the M1/M2 ratio is, the greater the overall survival rate and progression-free survival rate of patients [10, 11]. However, whether FPR2 has an influence on M2 macrophage differentiation is still controversial [12, 13].

In this study, RhoA expression was positively correlated with FPR2 in EOCs, and ectopic FPR2 expression promoted the migratory ability of EOCs, whereas an RhoA inhibitor (C3 transferase) diminished the migrational ability of EOCs. Moreover, FPR2 stimulated the secretion of Th2 cytokines by EOCs, which induced macrophages to differentiate to the M2 phenotype, while an RhoA inhibitor stimulated the secretion of Th1 cytokines and induced macrophages to differentiate to the M1 phenotype. Thus, we suggest that FPR2 stimulates M2 macrophage polarization and promotes invasion and metastasis of ovarian cancer cells through RhoA.

## Methods

### Cell cultures

The human ovarian cancer cell lines SKOV3, OVCAR3, A2780 and Caov3 were obtained from the America Type Culture Collection (ATCC; Manassas, VA, USA), and HO-8910 cell lines, Hosepic cell lines and THP-1 cell lines were obtained from the China Center for Type Culture Collection (CCTCC). The cell lines were cultured in either Dulbecco's modified Eagle's medium (DMEM, HyClone, Cat. No. SH30022.01B) or RPMI-1640 medium (HyClone, Cat. No. SH30809.01B) supplemented with 10% foetal bovine serum (FBS, HyClone, Cat. No. SH30256.01B) and antibiotics (penicillin 100 U/mL, streptomycin 0.1 mg/mL and amphotericin B 0.25 lg/mL) and maintained in a 37 °C incubator containing 5% CO_2_. THP-1 cells were treated with 10 ng/ml phorbol 12-myristate 13-acetate (PMA; Sigma Aldrich, Germany) for 72 h to induce differentiation of M0 macrophages. M0 macrophages were treated with 100 ng/ml LPS (#L2880, Sigma) plus 10 ng/ml IFN-γ (#BEK-2026, 4A Biotech, Beijing) for 48 h to induce M1 phenotype differentiation or with 10 ng/ml IL-4 (#214–14, PeproTech, Germany) for 48 h to induce M2 phenotype differentiation. Moreover, M0 macrophages were also treated with the supernatant of each ovarian cancer cell line for 48 h. Cells were collected for flow cytometry analysis, RT-qPCR or Western blot assays.

### Real-time quantitative PCR

Total RNA was extracted from cells using TRIzol reagents (Pufei Biotechnology, Shanghai, China). Reverse transcription was performed using M-MLV reverse transcriptase (Promega, Madison, USA).

The primer sequences were designed as follows:

FPR2: forward 5’-TTTGGCTGGTTCCTGTGTAAG-3’, reverse 5’-GGTCCGACGATCACCTTCAT-3’; RhoA: forward 5’-TGGATGGAAAGCAGGTAGAGT-3’, reverse 5’-CTATCAGGGCTGTCGATGGA-3’; and 18 s forward 5’-CCTGGATACCGCAGCTAGGA-3’, reverse 5’-GCGGCGCAATACGAATGCCCC-3’.

Quantitative PCR was performed using SYBR-Green RealTime PCR Master Mix (Toyobo, Osaka, Japan) according to the manufacturer's protocol. Data were analysed by Sequence Detection Software for the threshold cycle (Ct), and the comparative Ct (ΔΔCt) was used to calculate the difference between samples by relative qualification.

### Western blot analysis

Total cell lysates were harvested in NP-40 lysis buffer (150 mM NaCl, 1% Nonidet P-40, 50 mM Tris, pH 8.0, protease inhibitor cocktail), and protein concentrations were determined by BCA protein assay (Bio-Rad Laboratories, Inc., Hercules, CA, USA). Equal amounts of proteins from each lysate were submitted to SDS-PAGE for protein separation and then transferred to PVDF membranes. Membranes were blocked with buffer containing 5% skim milk and 0.1% Tween-20 in PBS for 1 h at room temperature with gentle shaking. Primary antibodies (FPR2, RhoA and GAPDH) were incubated overnight at 4 °C with gentle shaking, followed by secondary antibody incubation at room temperature for 1 h with gentle shaking. The following antibodies were used: FPR2 (#sc-66898; Santa Cruz Biotechnology, Santa Cruz, CA, USA); RhoA (#ab54835; Abcam, Cambridge, MA, USA); GAPDH (#KC-5G5; AKsomics, Shanghai); goat antirabbit IgG (H + L), and ads-HRP (#4050–05; Southern Biotech, Birmingham, AL, USA).

### Vector construction and plasmid transfection

The PCR product and pcDNA3.1 vector (Invitrogen) were both treated by double digestion of XhoI and HindIII. Target fragments were separated and purified, and the recombinant plasmid was constructed. T4-DNA ligase was used to combine the vector and the target gene. Caov-3 cell lines and OVCAR-3 cell lines were transfected with pcDNA3.1-shFPR2 vector and pcDNA3.1-FPR2 + vector, respectively, and both of the corresponding control cell lines were constructed. The shRNA sequences for FPR2 knockdown were as follows: shRNA-, ccggGGCCAAGACTTCCGAGAGAGActcgagTCTCTCTCGGAAGTCTTGGCCtttttg. The FPR2-overexpressing RNA sequence was TCACCTCCTGCAGAGACTGAGTTACAGGCAATGTGA.

### ELISA

The concentrations of cytokines, including TGF-β, IL-4, and IL-10, were determined using ELISA kits from Solarbio (Cat. No. #SEKH0316, #SEKH0011, and #SEKH0018, respectively). Cell culture supernatants were collected and centrifuged at 4 °C at 1000 × *g* for 10 min before analysis according to the manufacturer's protocol.

### Flow cytometry

For flow cytometric analysis, cells were stimulated as described above. A total of 1 × 10^6^ cells per sample were collected and stained with antibody in 100 µl PBS + 1% BSA for 30 min at 4 °C in the dark, followed by washing with PBS. Fluorescence was detected on a BD FACSCan (Becton, Dickinson and Company, MD, USA). All antibodies, except Dectin-PE (R&D Systems, Germany), were obtained from BD Bioscience (New Jersey, USA).

### Wound-healing assay

Cells (3 × 10^4^/well) were seeded on 96-well plates and grown to 90% confluence, after which a scratch was made in the monolayer using a 10-μl pipette tip. Then, the cells were incubated at 37 °C in 5% CO_2_ for another 4 h according to the results of the pre-experiment, and images were obtained at different time points. We captured the images for OVCAR3 cell lines at 0, 8 and 24 h, and for Caov3 cell lines, the time points were 0, 4 and 8 h. Each experiment was performed three times.

### Transwell assay

The assay was performed using a precoated cell invasion kit (pore size, 8.0 μm; Corning, Inc., Corning, NY, USA), and Matrigel (BD Biosciences, Bedford, MA, USA) was inserted into the upper chambers. Approximately 1 × 10^5^ cells in 100 μl serum-free medium were placed into the upper chambers, and the cells were cultured in 5% CO_2_ at 37 °C for 16 h (according to the pre-experiment). The lower chambers contained 30% FBS; thus, the cells migrated to the lower chambers. The cells remaining in the upper chambers were removed with a cotton swab, and the cells that migrated through the membrane to the lower surface were stained with Giemsa's staining for 3–5 min at room temperature. The number of cells that migrated through the lower membrane of the inserts was counted under a light microscope. Each experiment was performed three times.

### Xenografting

For in vivo establishment of tumour xenografts, 4-week-old female BALB/c nude mice (Linchang Biotechnology, Shanghai, China) were inoculated subcutaneously with SKOV3 and FPR2 knockdown SKOV3 cells (1 × 10^7^), and each group had 6 female BALB/c nude mice. Tumour sizes and tumour weights were measured on Days 30, 33, 36, 39, 42, 45, and 47 after subcutaneous inoculation, and the mice were euthanized by cervical dislocation on Day 47. Tumour sizes were measured in two dimensions, L and W, which represented the long and short diameters of the tumour, respectively. The volume (V) was expressed in mm^3^ with the formula: V = 3.14/6xLxW^2^. The animal work was approved by the Institutional Animal Care and Use Committee of Guangzhou Medical University and was conducted in accordance with the Guide for the Care and Use of Laboratory Animals (NIH Publication 85–23, revised 1996).

### Statistical analysis

Statistical analysis was performed using IBM SPSS Statistics 23.0 (IBM SPSS, Armonk, NY, USA). Statistics of continuous data were performed using ANOVA or the Kruskal–Wallis test; correlation analyses were performed using Pearson correlation analysis. At least three independent experiments for each group were conducted, and differences between groups were assessed by variance analysis and Student's t-test. A P value < 0.05 was considered to indicate a statistically significant result.

## Results

### FPR2 and RhoA were upregulated and positively correlated in EOC cell lines

**Fig. 1:**
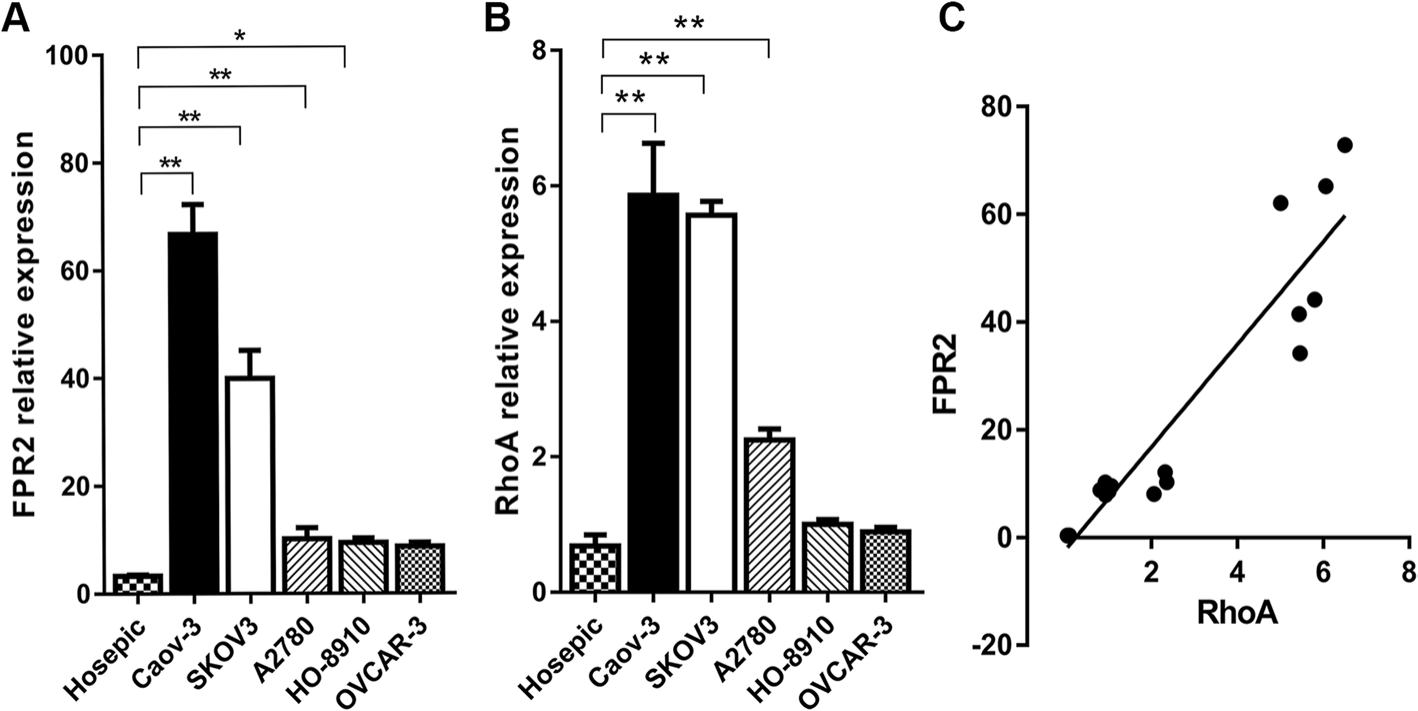
(A) FPR2 mRNA was upregulated in EOCs compared with the normal ovarian cell line (*P < 0.05), (**P < 0.01). (B) RhoAmRNA was upregulated in EOCs compared with the normal ovarian cell line (**P < 0.01). (C) Positive correlation between FPR2 and RhoA. The Pearson correlation coefficient was 0.936

First, we used RT-qPCR to examine the expression of FPR2 and RhoA in five EOC cell lines (Caov-3, SKOV3, A2780, HO-8910, OVCAR-3) and one normal ovarian epithelial cell line (Hosepic). We found that FPR2 and RhoA were both significantly highly expressed in EOCs compared with Hosepic cells. The Caov-3 cell line showed the highest expression compared with other EOC cell lines. Subsequently, we analysed the correlation between FPR2 and RhoA by Pearson correlation analysis, and the results showed that FPR2 expression was positively correlated with RhoA at the mRNA expression level. (Fig. 1A, B and C).

### RhoA expression changed along with the expression of FPR2

**Fig. 2:**
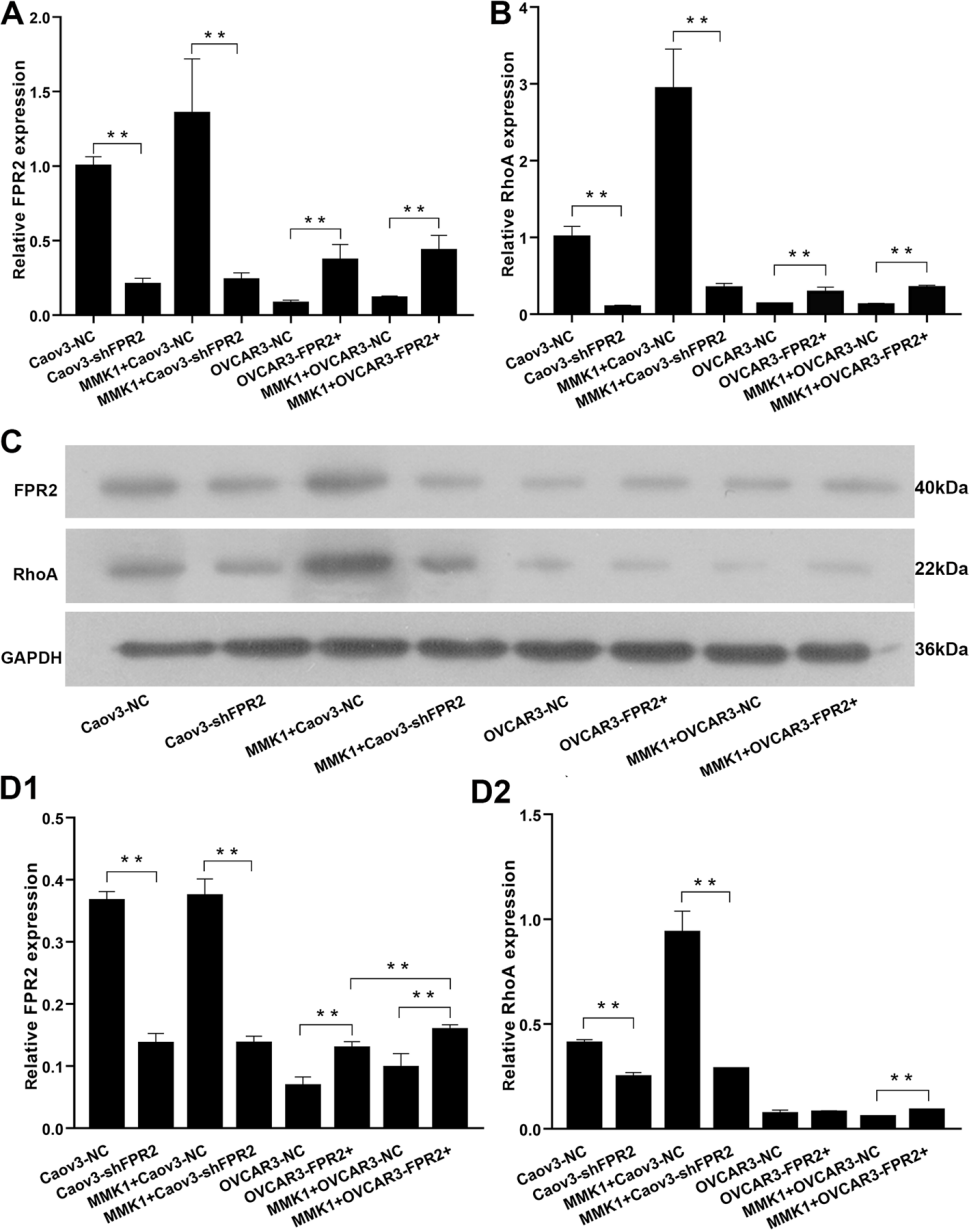
(A, B) RT-qPCR showed that FPR2 mRNA and RhoAmRNA were significantly increased in OVCAR3^−FPR2+^ cell lines and decreased in Caov3^−shFPR2^ cell lines regardless of MMK-1 treatment (**P < 0.01). (C, D1) Western blot analysis showed that FPR2 protein was significantly increased in OVCAR3^−FPR2+^ cell lines and decreased in Caov3-shFPR2 cell lines and was remarkably increased after stimulation with MMK-1 in OVCAR3^−FPR2+^ cell lines (**P < 0.01). (C, D2) Western blot analysis showed that RhoA protein was significantly decreased in Caov3^−shFPR2^ cell lines but showed no statistical significance in the OVCAR3^−FPR2+^ group and the control group, whereas RhoA was significantly increased in OVCAR3^−FPR2+^ cell lines when stimulated with MMK-1 (**P < 0.01)

To further study the role of FPR2 in RhoA expression, we established an FPR2-overexpressing OVCAR3 cell line (OVCAR3^−FPR2+^) and an FPR2-knockdown Caov3 cell line (Caov3^−shFPR2^), as well as their control cell lines (OVCAR3^−NC^, Caov3^−NC^). We treated each EOC with MMK-1 (a potential and selective FPR2 agonist) for 24 h. Then, RT-qPCR and Western blotting were performed to detect FPR2 and RhoA expression. RT-qPCR results showed that FPR2 mRNA was remarkably increased in the OVCAR3^−FPR2+^ cell lines and decreased in the Caov3^−shFPR2^ cell lines compared with their respective control groups. After stimulation with MMK-1, FPR2 mRNA was also increased in OVCAR3^−FPR2+^ cell lines compared with MMK1-treated OVCAR3^−NC^ cells. MMK-1 had no effect on FPR2 knockdown cells (Fig. 2A). RhoA expression was upregulated in OVCAR3^−FPR2+^ cell lines and downregulated in Caov3^−shFPR2^ cell lines compared with their respective control groups and significantly increased after stimulation with MMK-1 in OVCAR3^−FPR2+^ cell lines compared with MMK1-stimulated OVCAR3^−NC^ cells (Fig. 2B). Western blot results also showed that FPR2 protein was remarkably increased in the OVCAR3^−FPR2+^ cell lines and decreased in the Caov3^−shFPR2^ cell lines compared with their respective control groups, either after stimulation with MMK-1 or without treatment (Fig. 2C, D1). RhoA expression was also prominently decreased in Caov3^−shFPR2^ cell lines regardless of MMK-1 stimulation. In OVCAR3^−FPR2+^ cells, RhoA was highly expressed compared with its control group after stimulation with MMK-1 (Fig. 2C, D2).

### FPR2 and RhoA inhibitor (C3 transferase) had an adverse role in the motility of ovarian cancer cells

**Fig. 3:**
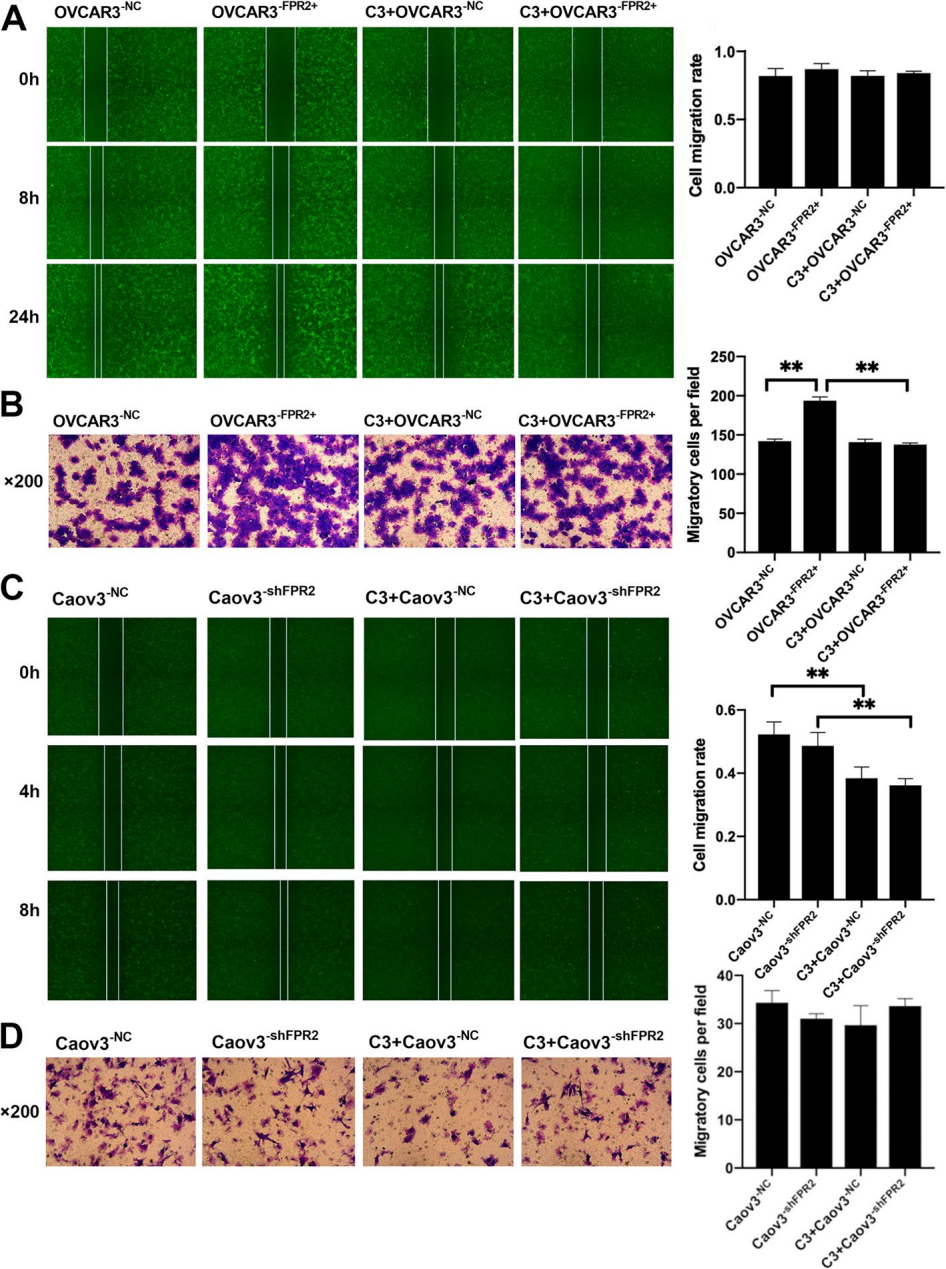
(A) The wound healing assay showed no significant difference in the cell migration rate in each group. (B) The Transwell assay showed that the number of transmembrane cells was evidently increased in OVCAR3^−FPR2+^ cell lines compared with OVCAR3^−NC^ cell lines. Compared to the number of transmembrane cells in OVCAR3^−FPR2+^ cell lines, the number of transmembrane cells was significantly increased in C3 + OVCAR3^−NC^ cell lines (**P < 0.01). (C) The wound healing assay showed that the cell migration rate was significantly decreased in C3 + Caov3^−NC^ cell lines compared with Caov3^−NC^ cell lines (**P < 0.01). Compared to Caov3^−shFPR2^ cell lines, the cell migration rate was also significantly deceased in C3 + Caov3^−shFPR2^ cell lines (**P < 0.01). (D) The number of migratory cells showed no significance in each group (*P* > 0.05)

Wound healing and Transwell assays were performed to clarify the migratory ability of EOCs. The wound healing assay showed that the average migration rate was not significantly different in each group of OVCAR3 cell lines (Fig. 3A). Transwell assays revealed that the number of EOCs that penetrated the Matrigel was significantly increased in the OVCAR3^−FPR2+^ group compared to the OVCAR3^−NC^ group. Moreover, the number of cells was significantly decreased in the OVCAR3^−FPR2+^ group after treatment with C3 transferase (Fig. 3B). Wound healing assays revealed that the migration rate was significantly decreased after treatment with C3 transferase in both Caov3^−NC^ cell lines and Caov3^−shFPR2^ cell lines (Fig. 3C). The number of transmembrane cells was decreased in Caov3^−shFPR2^ cell lines compared with the control group but showed no significant difference. After treatment with C3 transferase, the number of transmembrane cells was slightly decreased in C3 + Caov3^−NC^ cell lines compared with the Caov3^−NC^ cell lines, and C3 transferase had no effect in FPR2 knockdown Caov3 cell lines (Fig. 3D).

### The role of FPR2-stimulated Th2 cytokine secretion in EOC cells

**Fig. 4:**
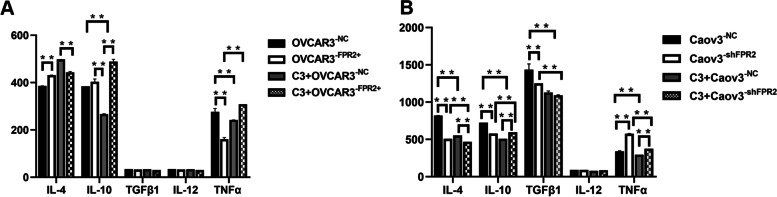
(A) IL-4 expression was significantly increased in OVCAR3^−FPR2+^ cell line supernatant compared with its control group (**P < 0.01), and IL-10 expression was significantly decreased in C3 + OVCAR3^−NC^ cell lines compared with OVCAR3^−NC^ cell lines (**P < 0.01). TNFα expression was significantly decreased in OVCAR3^−FPR2+^ cell line supernatant compared with its control group (**P < 0.01), and TNFα expression was also significantly decreased in OVCAR3 cell lines treated with C3 transfection (**P < 0.01). TGF-β1 and IL-12 expression showed no significant difference in each group (*P* > 0.05). (B) IL-4, IL-10 and TGF-β1 expression was significantly decreased in Caov3^−shFPR2^ cell line supernatants compared with the control group (**P < 0.01), and C3 transfection evidently decreased the secretion of IL-4, IL-10 and TGF-β1 in Caov3 cell lines (**P < 0.01). TNFα expression was significantly increased in the supernatant of Caov3^−shFPR2^ cell lines compared with its control group (**P < 0.01), and C3 transferase decreased TNFα secretion in Caov3 cell lines (**P < 0.01). IL-12 expression was not significantly different between the groups (*P* > 0.05)

The expression of TGF-β1, IL-4, IL-10, IL-12 and TNFα in EOC cell supernatant collected from each group was examined by ELISA kits. The results showed that IL-4 and IL-10 expression was higher in the supernatant of OVCAR3^−FPR2+^ cell lines than in the control group, and IL-10 secretion of OVCAR3 cell lines was distinctly reduced after treatment with C3 transferase. TNFα expression was significantly lower in the supernatant of OVCAR3^−FPR2+^ cell lines than in the control group, and C3 transferase increased TNFα secretion in OVCAR3^−FPR2+^ cell lines. IL-4, IL-10 and TGF-β1 expression was significantly lower in Caov3^−shFPR2^ cell supernatant than its control group, and C3 transferase diminished the secretion of IL-4, IL-10 and TGF-β1 in Caov3 cell lines. TNFα expression was evidently increased in the supernatant of Caov3^−shFPR2^ cell lines compared with its control group, and C3 transferase decreased TNFα secretion in Caov3 cell lines. (Fig. 4A, B).

### The effects of FPR2 on macrophage polarization

**Fig. 5:**
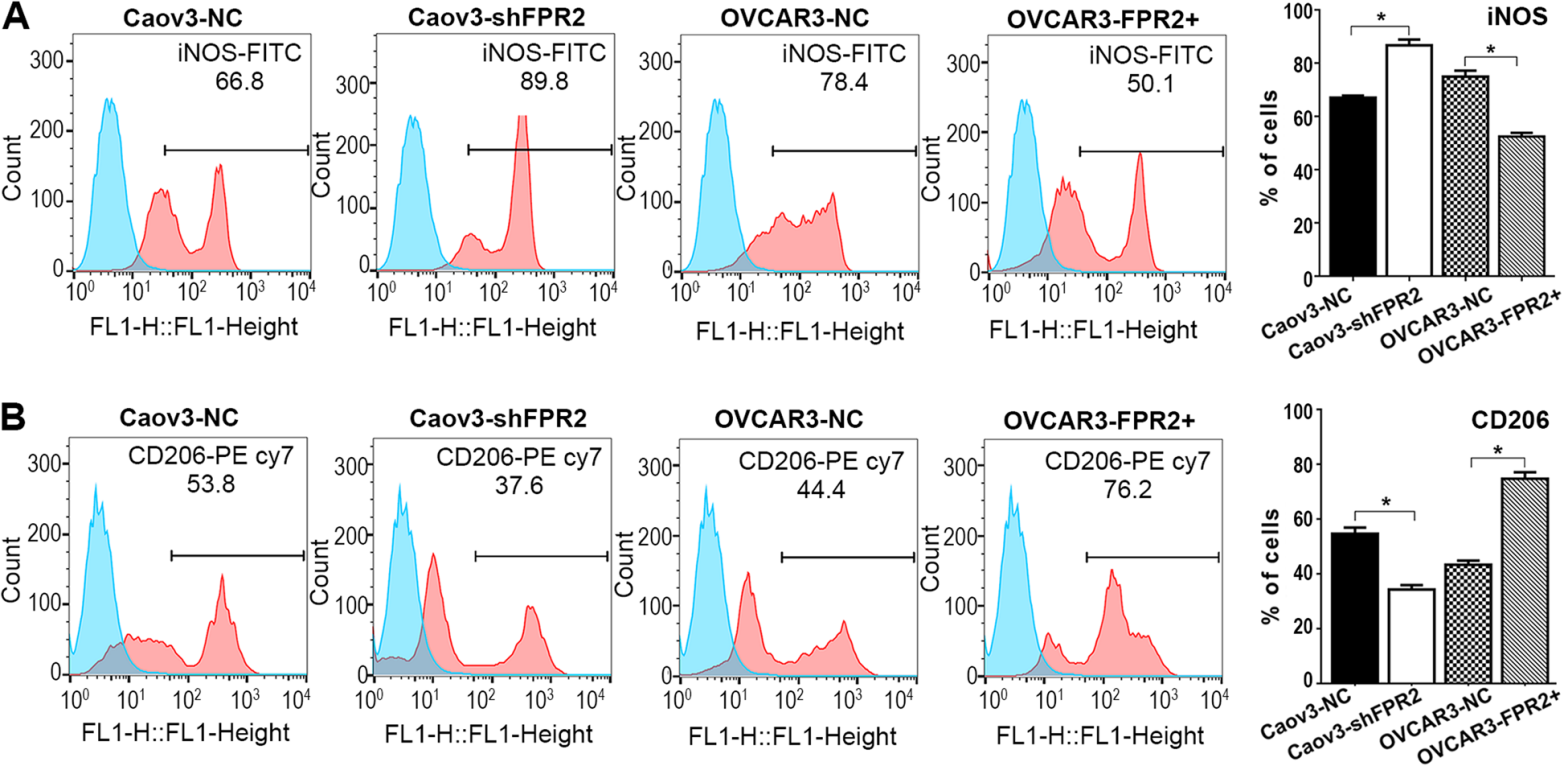
(A) The flow cytometry results showed that iNOS expression was significantly increased in the Caov3^−shFPR2^ group compared with the control group and was significantly decreased in the OVCAR3^−FPR2+^ group compared with the control group (*P < 0.05). (B) CD206 expression was significantly increased in the OVCAR3^−FPR2+^ group compared with the control group and was significantly decreased in the Caov3^−shFPR2^ group compared with the control group (*P < 0.05)

As reported, polarization of M2 macrophages is induced by Th2 cytokines. We treated macrophages with the cell supernatant of each cell line that we mentioned above. Subsequently, we detected M1 (iNOS) and M2 (CD206) markers in macrophages by flow cytometry. The results showed that iNOS expression was significantly upregulated in the Caov3^−shFPR2^ group and was downregulated in the OVCAR3^−FPR2+^ group; conversely, CD206 expression was significantly upregulated in the OVCAR3^−FPR2+^ group and was downregulated in the Caov3^−shFPR2^ group. (Fig. 5A, B).

### FPR2 and RhoA expression on M1 and M2 macrophages

**Fig. 6:**
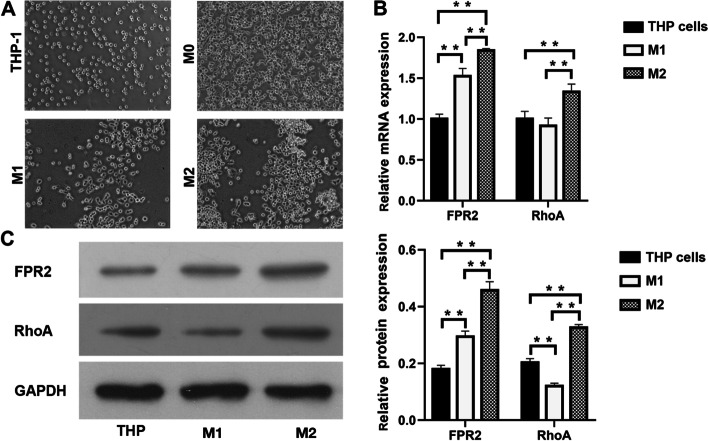
(A) THP-1 cells, M0, M1 and M2 macrophages. (B) RT-qPCR results showed that FPR2 mRNA expression and RhoAmRNA expression were both significantly increased in M2 macrophages compared to THP-1 cells and M1 macrophages (**P < 0.01). (C) Western blot results showed that relative FPR2 and RhoA protein expression were both evidently upregulated in M2 macrophages compared with THP-1 cells and M1 macrophages (**P < 0.01), and the expression of RhoA protein was significantly increased in M1 macrophages compared with THP-1 cells (**P < 0.01)

We also examined the expression of FPR2 and RhoA on macrophages of different phenotypes. THP-1 cells were induced to differentiate into M0 macrophages by PMA, and then M0 macrophages were induced to differentiate into M1 or M2 macrophages by LPS + IFN-γ or IL-4, respectively. Flow cytometry was performed to detect biomarkers of different phenotypes of macrophages, and RT-qPCR and Western blotting were used to detect the expression of FPR2 and RhoA in the THP-1 cell line and M1 and M2 macrophages, respectively. The results showed that FPR2 mRNA expression increased significantly in M1 and M2 macrophages compared with THP-1 cells, and M2 macrophages showed higher expression than M1 macrophages (Fig. 6B). RhoAmRNA was significantly upregulated in M2 macrophages compared with THP-1 cells and M1 macrophages, and no significant difference was shown between THP-1 cells and MI macrophages (Fig. 6B). Western blot results showed that FPR2 protein was significantly upregulated in M1 and M2 macrophages compared to the THP-1 cell line; RhoA protein expression was significantly decreased in M1 macrophages compared with THP-1 cells and increased in M2 macrophages compared with the THP-1 cell line and in M1 macrophages. (Fig. 6C).

### FPR2 contributed tumourigenic in vivo

**Fig. 7:**
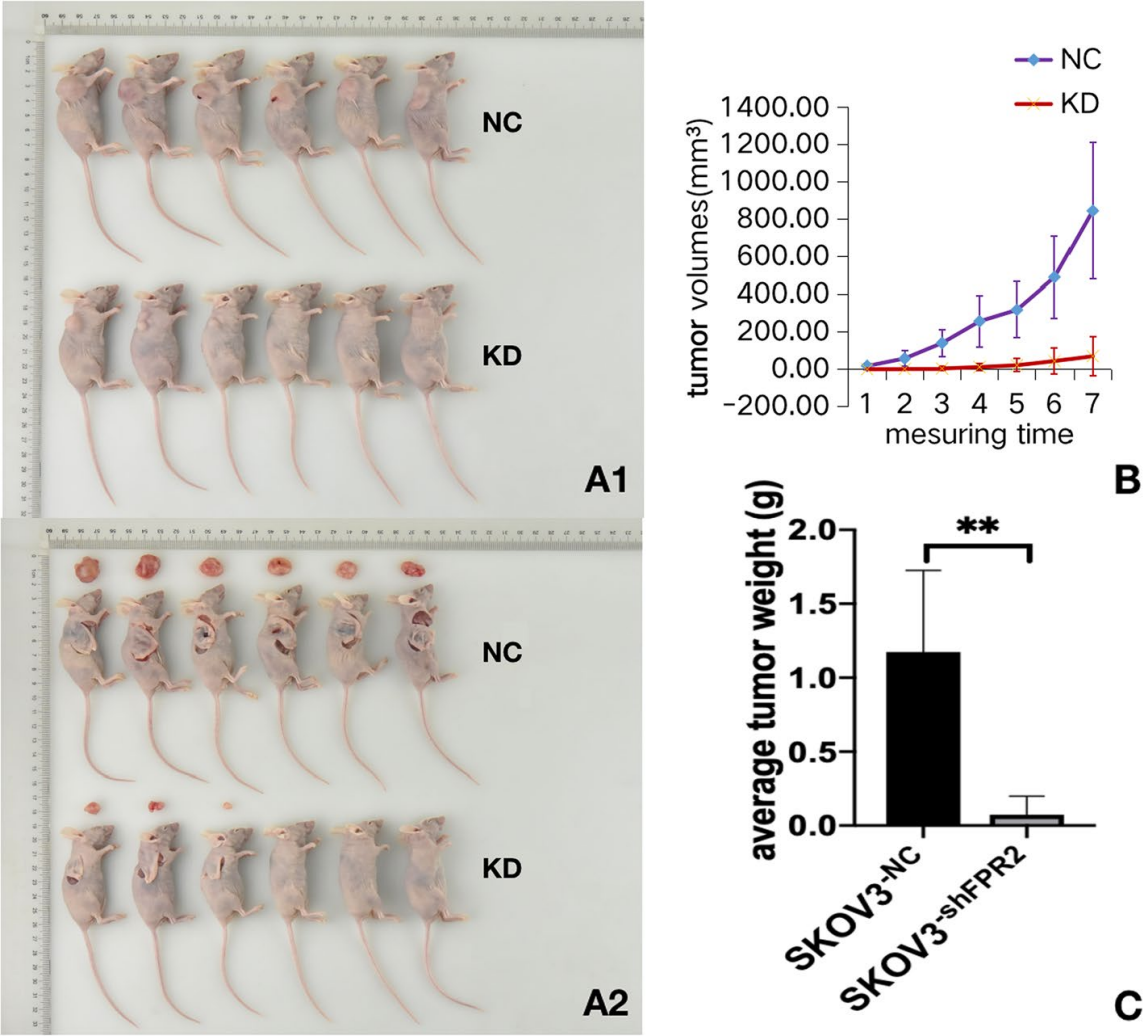
(A) Xenografts were inoculated subcutaneously with SKOV3 cell lines (NC group) and SKOV3^−shFPR2^ cell lines (KD group). (B) Tumour volumes showed a significant upwards trend in the NC group compared to the FPR2 knockout group. (B) The average tumour weight on Day 47 after subcutaneous inoculation was significantly heavier in the FPR2 knockout group than in the NC group (**P < 0.01)

To illustrate the role of FPR2 in vivo, we established tumour xenografts inoculated subcutaneously with SKOV3 cell lines (NC group) and SKOV3^−shFPR2^ cell lines (KD group) and then recorded and analysed the tumour volumes and tumour weights in each group. The average volumes of tumours in the NC group were 17.70 ± 13.88 mm^2^, 57.99 ± 40.59 mm^2^, 140.85 ± 73.13 mm^2^, 257.35 ± 136.83 mm^2^, 318.79 ± 150.46 mm^2^, 491.18 ± 217.31 mm^2^, and 846.09 ± 365.02 mm^2^ on Days 30, 33, 36, 39, 42, 45, and 47, respectively. In the KD group, the average tumour volumes were 0.39 ± 0.95 mm^2^, 1.33 ± 3.26 mm^2^, 4.14 ± 10.14 mm^2^, 11.72 ± 20.62 mm^2^, 44.04 ± 71.58 mm^2^, and 70.03 ± 105.58 mm^2^, respectively. The average weights of tumours in the NC group and KD group were 1.116 ± 0.514 g and 0.137 ± 0.191 g, respectively. The results showed that the tumour volumes and weights were significantly decreased after FPR2 knockdown, which suggested that FPR2 may play a role in ovarian cancer tumourigenesis (Fig. 7A1,A2,B and C).

## Discussion

In this study, we first demonstrated the interactions between FPR2 and RhoA in epithelial ovarian cancer cells. We found that RhoA expression in EOCs was increased with the overexpression of FPR2, whereas with the knockdown of FPR2 in EOCs, RhoA expression was decreased correspondingly. FPR2 is one of the members of the formyl peptide receptor family, which belongs to the G-protein coupled receptor (GPCR) family [14]. GPCRs play a key role in regulating the sensitivity to chemokines, and the signaling of other GPCRs involved in migration and migration requires the coordinated activation of hundreds of proteins in distinct compartments of the cell [15]. The Rho GTPase family is part of the Ras superfamily, and Rho GTPases are highly conserved and found in nearly all leukaryotes, acting as molecular switches and cycling between an active GTP-bound form and an inactive GDP-bound form. GPCRs are activated through binding to intracytoplasmic Rho proteins and stimulate downstream signal transduction. They contribute to several cellular processes and pathological processes, including cell morphogenesis, cell polarity or migration, cancer progression, inflammation and wound repair [16, 17].

These results suggest that FPR2 mediates the activation of Rho proteins and affects downstream signal transduction. According to the study by Faour W.H. et al., the FPR2 agonist fMLP activated the ERK1/2 and Akt pathways through specific activation of the FPR2/ROS/RhoA-GTPase pathway and stimulated H_2_O_2_ release by monocytes [6]. In bone marrow PMNs, the RhoA/ROCK pathway was activated in the respiratory burst via mFPR1 and mFPR2, RhoA was considered to be one of the regulatory and signal transduction components in the respiratory burst through FPRs, and both mFPR1 and mFPR2 binding with a ligand triggered the activation of RhoA and regulated NADPH oxidase activity [7]. In this study, we preliminarily clarified the positive correlation of FPR2 and RhoA in ovarian cancer cells, and a RhoA inhibitor reversed the migration ability of EOCs, which was promoted by ectopic expression of FPR2. Further studies revealed that secretion of Th2 cytokines was increased by ovarian cancer cells with high FPR2 expression, which induced M2-like macrophage polarization, and C3 transferase partly inhibited the polarization of M2 macrophages.

Macrophages play an indispensable role in defending against microbial infections and tumour cells. Tumour-associated macrophages (TAMs) constitute a significant proportion of tumour-infiltrating immune cells, predominantly resemble M2-like polarized macrophages and produce a high amount of anti-inflammatory factors that contribute to the development of tumours [18]. Interferon regulatory Factor 5 (IRF5) and IRF4 have been reported to act as two key transcriptional regulators in regulating the polarization of macrophages to the M1 and M2 phenotypes, respectively. IRF5 expression drives M1 macrophage polarization by directly inducing the expression of proinflammatory cytokines and repressing the transcription of anti-inflammatory cytokines such as IL-10. In contrast, IRF4 has been shown to be a crucial mediator of M2 macrophage polarization [19]. Moreover, the status of macrophage polarization can also be polarized or reversed by cellular signalling pathways. JNK signalling pathway activation induces macrophage polarization to the M2 phenotype, while activation of the Notch signalling pathway promotes M1 macrophage polarization [20].

IL-10 is an anti-inflammatory cytokine that plays a critical role in the control of immune responses in both inflammation and cancer. IL-10 is a cytokine whose levels have been shown to be elevated in the tumour microenvironment and blood of tumour-bearing mice as well as ovarian cancer patients. IL-10 also acts as a critical regulator of the PD-1/PD-L1 axis for immunosuppression in the ovarian tumour microenvironment [21]. Studies have shown that IL-10 regulates the metabolic processes of glycolysis and oxidative phosphorylation in macrophages and inhibits the switch to the metabolic program induced by inflammatory stimuli in macrophages [22]. In lung cancer, IL-10 is thought to suppress the inflammatory macrophage-Th17 cell axis, which is critical to tumourigenesis, and may be used to prevent lung cancer in high-risk patients [23]. IL4/IL4R signalling acts as a prometastatic phenotype in epithelial cancer cells, including enhanced migration, invasion, survival, and proliferation. Studies have revealed that IL-4 antibody neutralization enhances antitumour immunity and delays tumour progression. IL-4 blockade also alters inflammation in the tumour microenvironment, reducing the generation of both immunosuppressive M2 macrophages and myeloid-derived suppressor cells and enhancing tumour-specific cytotoxic T lymphocytes [24]. In our study, we found that FPR2 played an auxoaction on the secretion of IL-10 and IL-4 in ovarian cancer cells and the induction of M2 macrophage polarization. Currently, the effect of FPR2 on macrophage polarization is unclear. Studies have shown that FPR2 plays a critical role in antitumour host immunity by limiting macrophage recruitment into tumours and sustaining macrophages in an M1 phenotype [13]. In mice, FRP2 deletion reduces tissue and systemic inflammation by inhibiting macrophage infiltration and M1 polarization [25]. FPR2 was also considered a mediator that led to macrophage skewing in a model of skeletal muscle injury and repair, which accelerated muscle regeneration [26]. The results of this study demonstrated that FPR2 participated in M2 macrophage differentiation. M1 macrophages are generally accepted to be responsible for the stimulation of the immune system and inflammation, while M2 macrophages play a role in cancer and tissue repair. Our results showed that FPR2 was differentially expressed between M1 and M2 macrophages, which may indicate that FPR2 might play a role in both M1 and M2 macrophage phenotype and function, while RhoA might be inclined to induce an M2 macrophage phenotype, which still needs further study.

## Conclusions

In this study, RhoA expression was significantly increased in EOCs along with the overexpression of FPR2, which showed a positive correlation by Pearson correlation analysis. Moreover, FPR2 promoted the migratory ability of EOCs, and an RhoA inhibitor had an adverse effect on EOC migration. In addition, FPR2 stimulated the secretion of Th2 cytokines by EOCs, which induced macrophages to differentiate to the M2 phenotype, while an RhoA inhibitor stimulated the secretion of Th1 cytokines and induced macrophages to differentiate to the M1 phenotype. Therefore, we suggest that FPR2 stimulates M2 macrophage polarization and promotes invasion and metastasis of ovarian cancer cells through RhoA. Further study of the mechanisms of FPR2-regulated macrophage polarization and its role in ovarian cancer progression is needed.

## Data Availability

The datasets generated and analysed during the current study are available from the corresponding author on reasonable request.

## References

[1] Matulonis UA, Sood AK, Fallowfield L, Howitt BE, Sehouli J, Karlan BY (2016). Ovarian cancer Nat Rev Dis Primers.

[2] Bast RJ, Hennessy B, Mills GB (2009). The biology of ovarian cancer: new opportunities for translation. Nat Rev Cancer.

[3] Ye RD, Boulay F, Wang JM, Dahlgren C, Murphy PM (2009). International Union of Basic and Clinical Pharmacology. LXXIII. Nomenclature for the formyl peptide receptor (FPR) family. Pharmacol Rev.

[4] Li Y, Ye D (2013). Molecular biology for formyl peptide receptors in human diseases. J Mol Med (Berl).

[5] Xie X, Yang M, Ding Y, Yu L, Chen J (2017). Formyl peptide receptor 2 expression predicts poor prognosis and promotes invasion and metastasis in epithelial ovarian cancer. Oncol Rep.

[6] Faour WH, Fayyad-Kazan H, Zein NE (2018). fMLP-dependent activation of Akt and ERK1/2 through ROS/Rho A pathways is mediated through restricted activation of the FPRL1 (FPR2) receptor. Inflamm Res.

[7] Filina JV, Gabdoulkhakova AG, Safronova VG (2014). RhoA/ROCK downregulates FPR2-mediated NADPH oxidase activation in mouse bone marrow granulocytes. Cell Signal.

[8] Quail DF, Joyce JA (2013). Microenvironmental regulation of tumor progression and metastasis. Nat Med.

[9] Mills CD, Ley K (2014). M1 and M2 macrophages: the chicken and the egg of immunity. J Innate Immun.

[10] Yin M, Li X, Tan S, Zhou HJ, Min W (2016). Tumor-associated macrophages drive spheroid formation during early transcoelomic metastasis of ovarian cancer. J Clin Invest.

[11] Yuan X, Zhang J, Li D, Mao Ye, Mo F, Wei Du (2017). Prognostic significance of tumor-associated macrophages in ovarian cancer: A meta-analysis. Gynecol Oncol.

[12] Li Y, Cai L, Wang H, Wu P, Gu W, Chen Y (2011). Pleiotropic regulation of macrophage polarization and tumorigenesis by formyl peptide receptor-2. Oncogene.

[13] Liu Y, Chen K, Wang C, Gong W, Yoshimura T, Liu M (2013). Cell surface receptor FPR2 promotes antitumor host defense by limiting M2 polarization of macrophages. Cancer Res.

[14] Hilger D, Masureel M, Kobilka BK (2018). Structure and dynamics of GPCR signaling complexes. Nat Struct Mol Biol.

[15] Cleghorn WM, Bulus N, Kook S, Gurevich VV, Zent R, Gurevich EV (2018). Non-visual arrestins regulate the focal adhesion formation via small GTPases RhoA and Rac1 independently of GPCRs. CELL SIGNAL.

[16] Haga RB, Ridley AJ (2016). Rho GTPases: Regulation and roles in cancer cell biology. Small GTPases.

[17] Hodge RG, Ridley AJ (2016). Regulating Rho GTPases and their regulators. Nat Rev Mol Cell Biol.

[18] Sica A, Larghi P, Mancino A, Rubino L, Porta C, Totaro MG (2008). Macrophage polarization in tumour progression. Semin Cancer Biol.

[19] Krausgruber T, Blazek K, Smallie T, Alzabin S, Lockstone H, Sahgal N (2011). IRF5 promotes inflammatory macrophage polarization and TH1-TH17 responses. Nat Immunol.

[20] Cheng H, Wang Z, Fu L, Xu T (2019). Macrophage Polarization in the Development and Progression of Ovarian Cancers: An Overview. FRONT ONCOL.

[21] Lamichhane P, Karyampudi L, Shreeder B, Krempski J, Bahr D, Daum J (2017). IL10 Release upon PD-1 Blockade Sustains Immunosuppression in Ovarian Cancer. CANCER RES.

[22] Ip W, Hoshi N, Shouval DS, Snapper S, Medzhitov R (2017). Anti-inflammatory effect of IL-10 mediated by metabolic reprogramming of macrophages. Science.

[23] Li Q, Anderson CD, Egilmez NK (2018). Inhaled IL-10 Suppresses Lung Tumorigenesis via Abrogation of Inflammatory Macrophage-Th17 Cell Axis. J Immunol.

[24] Ito SE, Shirota H, Kasahara Y, Saijo K, Ishioka C (2017). IL-4 blockade alters the tumor microenvironment and augments the response to cancer immunotherapy in a mouse model. Cancer Immunol Immunother.

[25] Chen X, Zhuo S, Zhu T, Yao P, Yang M, Mei H (2019). Fpr2 Deficiency Alleviates Diet-Induced Insulin Resistance Through Reducing Body Weight Gain and Inhibiting Inflammation Mediated by Macrophage Chemotaxis and M1 Polarization. Diabetes.

[26] Machado MG, Tavares LP, Souza G, Queiroz-Junior CM, Ascencao FR, Lopes ME (2020). The Annexin A1/FPR2 pathway controls the inflammatory response and bacterial dissemination in experimental pneumococcal pneumonia. FASEB J.

